# Smoking cessation in pregnancy: An update for maternity care practitioners

**DOI:** 10.18332/tid/109906

**Published:** 2019-08-02

**Authors:** Athina Diamanti, Sophia Papadakis, Sotiria Schoretsaniti, Nikoletta Rovina, Victoria Vivilaki, Christina Gratziou, Paraskevi A. Katsaounou

**Affiliations:** 1 School of Medicine, National and Kapodistrian University of Athens, Athens, Greece; 2 Department of Midwifery, University of West Attica, Athens, Greece; 3 Division of Prevention and Rehabilitation, University of Ottawa Heart Institute, Ottawa, Canada; 4 Center for Health Services Research, Department of Hygiene, Epidemiology and Medical Statistics, School of Medicine, National and Kapodistrian University of Athens, Athens, Greece; 5 1st Department of Respiratory Medicine, ‘Sotiria’ Chest Disease Hospital, Athens, Greece; 6 Evgenidio Hospital, Athens, Greece; 7 First ICU, Evangelismos Hospital, Athens, Greece

**Keywords:** pregnancy, smoking cessation, secondhand smoke, nicotine replacement therapy, review

## Abstract

**INTRODUCTION:**

This paper provides an up-to-date summary of the effects of smoking in pregnancy as well as challenges and best practices for supporting smoking cessation in maternity care settings.

**METHODS:**

We conducted a qualitative review of published peer reviewed and grey literature.

**RESULTS:**

There is strong evidence of the effects of maternal tobacco use and secondhand smoke exposure on adverse pregnancy outcomes. Tobacco use is the leading preventable cause of miscarriage, stillbirth and neonatal deaths, and evidence has shown that health effects extend into childhood. Women who smoke should be supported with quitting as early as possible in pregnancy and there are benefits of quitting before the 15th week of pregnancy. There are a variety of factors that are associated with tobacco use in pregnancy (socioeconomic status, nicotine addiction, unsupportive partner, stress, mental health illness etc.). Clinical-trial evidence has found counseling, when delivered in sufficient intensity, significantly increases cessation rates among pregnant women. There is evidence that the use of nicotine replacement therapy (NRT) may increase cessation rates, and, relative to continued smoking, the use of NRT is considered safer than continued smoking. The majority of women who smoke during pregnancy will require support throughout their pregnancy, delivered either by a trained maternity care provider or via referral to a specialized hospital or community quit-smoking service. The 5As (Ask, Advise, Assess, Assist, Arrange) approach is recommended for organizing screening and treatment in maternity care settings. Additionally, supporting smoking cessation in the postpartum period should also be a priority as relapse rates are high.

**CONCLUSIONS:**

There have been several recent updates to clinical practice regarding the treatment of tobacco use in pregnancy. It is important for the latest guidance to be put into practice, in all maternity care settings, in order to decrease rates of smoking in pregnancy and improve pregnancy outcomes.

## INTRODUCTION

Maternal tobacco smoking and secondhand smoke (SHS) exposure during pregnancy are the leading preventable causes for a variety of unfavorable pregnancy outcomes and continue to be a major public health concern^1,2^.

Available data suggest that 15–20% of all pregnant women will continue to smoke during pregnancy^3-6^. Smoking in pregnancy has decreased in high-income countries but is still increasing in low-to-middle-income countries^7^. In countries such as the United States, Denmark and Sweden, the prevalence of smoking in pregnancy has declined from between 20% and 35% in the 1980s to between 10% to 20% in the 2000s, and below 10% by 2010^1^. However, the decline has not been consistent across all sectors of society, with lower rates of decline in lower socioeconomic groups. National rates of smoking in pregnancy appear to be associated with economic development; e.g. in Poland the prevalence is estimated at 30%, while the prevalence in countries such as the Democratic Republic of Congo is still very low. However, given the aggressive nature of tobacco marketing, there is concern that prevalence of smoking in pregnancy will increase with economic development, with subsequent health impacts on countries with already high disease burdens and limited resources to provide health care and in particular neonatal care^1^. In countries like Greece 48% of pregnant women still report smoking in the first trimester^3^. Many pregnant women will continue to be exposed to tobacco smoke, from other smokers around them, including partners, and in SHS environments^3-5,8,9^.

Supporting pregnant women with quitting smoking and addressing SHS exposure are two of the most significant interventions that can be employed by healthcare professionals in order to lower the risk of adverse birth outcomes. Healthcare professionals should be trained in the latest evidence-based practices in addressing tobacco use among pregnant women and be prepared to intervene in their clinical settings in order to achieve a higher percentage of smoking cessation among pregnant smokers^3-5,10-13^.

This paper provides an up-to-date summary of the adverse effects of smoking in pregnancy, factors and challenges associated with smoking cessation in pregnancy, and available evidence regarding best practices for smoking cessation in maternity care settings, with a focus on midwives and obstetricians.

## METHODS

We conducted a qualitative review of published peer reviewed and grey literature. We followed the following procedures. We searched MEDLINE/PUBMED between January 2010 and February 2019. The key words used were: ‘pregnancy’, ‘pregnant women’, ‘post-partum’, ‘smoking’, ‘smoking cessation’, and ‘tobacco’. We also searched the Cochrane Collaboration reviews and conducted an online search of grey literature (i.e. government commissioned and other reports). All papers and reports included were published in English and reported on one of the following topics of interest to this review: 1) the health effects of smoking in pregnancy on maternal, fetal or child health; 2) barriers and facilitators associated with smoking and quitting in pregnancy and the post-partum period; 3) the effectiveness of smoking cessation interventions among pregnant women; and 4) the safety of smoking cessation interventions in pregnancy. When available the most recent meta-analysis was used as the key reference.

## RESULTS

### Health effects of smoking in pregnancy

There is clear evidence of the strong association between maternal tobacco use and the increase of serious adverse pregnancy-related outcomes with effects extending into childhood^2,14,15^. Pregnancy related health effects of maternal smoking are summarized in Table 1^16^.

**Table 1:** Pregnancy related health effects of smoking

*Pregnancy related health effects of smoking*			
*Fertility*	*Obstetrics*	*Fetal*	*Offspring (child and adult)*
**Delayed conception**	**Spontaneous miscarriage**	**Growth restriction**	**SIDS**
**Female infertility** (60% increased risk)	**Premature rupture of membranes**	**Low birth weight**	**Respiratory** (Asthma, lower respiratory infection, Decreased lung function)
	**Ectopic pregnancy**	**Small for gestational age**	**Type 2 diabetes**
**Assisted reproduction** (44% reduced odds of a live birth cycle)	**Stillbirth**	**Birth defects** (Limb reduction, clubfoot, cleft lip or palate, anophthalmia, esotropia, microphthalmia, exotropia, optic nerve hypoplasia, heart defects, craniosynostosis, gastroschisis, anal atresia, hernia, cryptorchidism	**Neurologic & Neurodevelopmental effects** (developmental abnormalities in the brain, impairments in normal brain activity)
**Male infertility** (Decreased semen volume, sperm number, increased abnormal forms)	**Placental vascular resistance**		**Cognition** (Impaired academic performance and cognitive abilities, global intelligence/academic performance)
	**Placental abruption**		**Behavior** (ADHD, Conduct disorder, antisocial behavior, externalizing behavior)
	**Placenta previa**		**Nicotine dependence and future substance abuse**
	**GDM**		**Psychiatric disorders**
	**Preterm birth**		**Obesity**
			**Decreased HDL**
			**Hypertension**
			**NEC**
			**Reproduction** (earlier menarche for girls, reduction in male reproduction ability)

ADHD: Attention Deficit Hyperactivity Disorder, NEC: Necrotizing Entero Colitis, GDM: Gestation Diabetes Mellitus, SIDS: Sudden Infant Death Syndrome

Specifically, maternal smoking is associated with an increased risk of adverse obstetric effects^14^ and placental vascular resistance^2^, miscarriage^14^, preterm birth^14^, abruption placenta^14^, placental previa^14^, preterm rupture of the membranes^14^, ectopic pregnancy^14^, stillbirth^14^, gestation diabetes mellitus^17^ and fetal effects^14^, low birth weight^14^, small-for-gestational-age^14^, and Intra-Uterine Growth Retardation (IUGR)^14^. There is increased risk also of birth defects^2^ including: limb reduction defects^14,17,18^, clubfoot^14,18^, cleft lip or palate^14,18^, anophthalmia^14,18^, microphthalmia^14,18^, esotropia^18^, exotropia^18^, optic nerve hypoplasia^18^, heart defects^14,18^, craniosynostosis^14,18^, gastroschisis^18^, anal atresia^14,18^, hernia^14,18^, and cryptorchidism^14,15,18^.

There is also evidence in terms of the adverse effects of maternal tobacco use extending to newborns and children. Namely, there is a significantly increased risk of sudden infant death syndrome^2,14^, obesity^2,14^, type 2 diabetes^2,14,17^, earlier menarche for girls^2^, and reduction in male reproduction ability^19^, respiratory effects (upper respiratory tract infections, bronchitis and other lower respiratory tract infections, pulmonary hypertension, compromised lung function, increased rate of lower respiratory tract infections, impaired lung function, wheezing and asthma^2,14^, cardiovascular effects (hypertension)^2,14^, neurologic effects (developmental abnormalities in the brain, decreased brain measurements, impairments in normal brain activity)^2^, neurodevelopmental and behavioral effects^2,14^ (abnormal behavioral and neurodevelopmental outcomes)^2^, global intelligence/ academic performance^2^, Attention Deficit/ Hyperactivity Disorder (ADHD)^2,20^, externalizing behaviors^2,20^, antisocial behavior, nicotine dependence^14^ and future substance abuse^2^, significant increase risk for early psychiatric disorders^21^ in early adulthood, and necrotizing enterocolitis (NEC)^2^.

Secondhand smoke exposure during pregnancy is associated with many of the same adverse pregnancy outcomes^10,22-24^.

### Mechanism of smoking effects on pregnancy outcomes

Nicotine and carbon monoxide (CO) are two main derivatives of tobacco that have a negative effect on pregnancy outcomes. Nicotine is a known neurotoxin and exposure to it interferes with normal neurotransmitter function, and when present in sufficient levels can be harmful to the developing fetus. In pregnancy, the developing fetus is exposed to higher nicotine levels than the smoking mother, as nicotine is concentrated in the fetal compartment^2^. Moreover, unlike many teratogens, it appears that nicotine is more harmful to the developing fetus during the latter part of pregnancy, with the third trimester being the most sensitive^2^. CO is a very toxic gas contained in cigarette smoke^11,25^. The CO that is inhaled when smoking results in the formation of carboxyhemoglobin (COHb), which adversely affects both mother and fetus since it reduces the oxygen carrying capacity in the blood and can lead to fetal hypoxia^11,25^.

Although quitting smoking at any stage of pregnancy is associated with improved pregnancy outcomes, there is evidence that quitting smoking in the first trimester of pregnancy provides the greatest benefits^26^. Specifically, women who quit smoking before the 15th week of pregnancy reduce the risk of a preterm birth and small-for-gestational-age babies to that of a non-smoker^11,26^. As such, quitting early in pregnancy should be a clinical priority.

### Barriers to quitting in pregnancy

There are many factors associated with smoking during pregnancy such as socioeconomic status (low educational attainment and deprivation), White race, higher level of nicotine dependence, lack of social support, having a partner who smokes or other smokers in the home, using alcohol during pregnancy, culture, high levels of stress, and mental health conditions including depression and past-year psychiatric symptoms^4,7,27^. Having a supportive partner is particularly important, as it can greatly help increase the ability of a pregnant smoker to quit successfully^4^. Pregnant women with partners who are active smokers find it harder to quit and are more likely to relapse, especially during the postnatal period^28^.

The biological and hormonal changes, which occur during pregnancy, can also make quitting challenging for some women. Specifically, nicotine metabolism increases during pregnancy and as a result pregnant women may experience an increase in both cravings and withdrawal symptoms when quitting^29^. Moreover, certain associations between smoking and daily routines, emotions, people, places, have been created that become cues for smoking and are part of the challenge to quitting^30^. Thus, pregnant women trying to quit smoking face a combination of physical and psychological addiction, which can make quitting smoking particularly challenging.

### Evidence-based smoking cessation interventions in pregnancy

#### Behavioral counseling

There is strong clinical-trial evidence to support that counseling can significantly increase rates of smoking cessation^8,31-33^. Available research and experience suggests that cessation counseling for pregnant women should be delivered at a sufficient intensity in order to increase efficacy^19^. Generally, counseling sessions are recommended to be at least 15 minutes in duration and be delivered by either a trained maternity care provider or via referral to a hospital or community-based quit-smoking service^30,31,33^. Given that relapse rates during pregnancy are high, counseling support should be delivered throughout the duration of the pregnancy.

There are several pieces of practical counseling advice that can be helpful to offer pregnant women. Specifically, women should be^3,5,8,9,34^:

- Informed of the significant effects of smoking on pregnancy outcomes;
- Informed of typical withdrawal symptoms, which can be quite intense during the first few weeks of tobacco abstinence and supported with developing coping strategies;
- Instructed to remove any tobacco related products from their environment; and
- Advised to avoid or reduce the amount of time spent with people, places or social situations that might have been strongly associated with smoking.

Table 2 presents a brief summary of strategies to be employed to support cessation during pregnancy as part of antenatal and postnatal care. All past quitting attempts (if any) should be revisited, to identify both factors that were helpful in supporting cessation as well as those that contributed to relapse.

**Table 2:** Interventions for SC in pregnancy

*Interventions for smoking cessation in pregnancy*
Emphasizing the negative health effects for both mom and baby, including effects of secondhand smoke exposure
Encourage a woman to ask support from her social network
Help her identify trigger factors so as to avoid withdrawals
Provide training so as to deal with or avoid triggers to smoke
Training on behavioral and mental coping skills
Discussing on ways they can spend the money saved by not buying cigarettes
Provide Information on weight gain
Shifting focus on the ‘new role’ as a mother and its responsibilities
Shifting focus to the motivation for quitting from extrinsic sources to intrinsic sources
Help the woman to establish a non-smoking support system
Support the women with positive encouragement rather than negative nagging
Reaffirm her commitment to abstinence
Pharmacotherapy
Include the smoking habits of partners, others living in the home, and close friends
Take place throughout pregnancy through early childhood care
Discuss the risks of relapse immediately after childbirth

Adapted from Campbell et al.^30^, Bauld et al.^28^, Riaz et al.^7^, Naughton et al.^69^, and McEwen^13^.

Two counseling techniques have been shown to have positive effects in supporting cessation among pregnant women. These are Cognitive Behavioral Therapy (CBT) and Motivational Interviewing (MI).

CBT is a counseling technique that has been shown to increase quit rates^31^. CBT is grounded in the assumption that people with disturbances in the functioning of their thoughts and emotions are more susceptible to becoming smokers and to continue being smokers. CBT aims to help individuals deal with problems in a more positive way by breaking them down into smaller parts. Individuals are shown how to change these negative patterns to improve the way they feel^9,33,35^. Specific aspects of CBT counseling that have been shown to benefit pregnant women include^31,33^:

- Developing a sense of self-monitoring, self-control, self-discipline and self-preservation;
- Identifying strategies for managing the cravings to smoke when they occur;
- Managing situations with stress and anxiety;
- Providing social support and boosting self-esteem and the sense of self-efficacy (confidence); and
- Goal setting and action planning.

Motivational Interviewing (MI) is a patient-centered, collaborative, goal-oriented method designed to strengthen personal motivation and commitment to a specific goal by eliciting and exploring the person’s own reasons for change within an atmosphere of acceptance and compassion^36^. MI has been found effective in supporting smoking cessation in pregnancy^9^, but further development and application of specific programs by nurses and midwives working in antenatal settings are needed as part of MI information; advice is offered with permission from the individuals, and the individuals autonomy for decision-making is respected. The goal for the health care providers is to understand the individual’s perspective on the topic and their needs, and to assist the individuals to draw their own conclusions about the relevance of any information provided^37^. The five principles of MI are: 1) Express Empathy, 2) Develop Discrepancy, 3) Avoid Argumentation, 4) Roll with Resistance, and 5) Support Self-efficacy (i.e. build patient confidence in their ability to achieve the change/goal).

#### Pharmacotherapy

There are three first-line quit smoking medications with proven efficacy for supporting cessation: nicotine replacement therapy (NRT), varenicline, and bupropion (Table 3).

**Table 3:** Pharmacotherapy

*Pharmacotherapy*
**NRTs**
Introduced as early as possible in pregnancy
Use the lowest dose that controls withdrawal symptoms and permits abstinence, and then increase dose if necessary
Short acting NRT products (e.g. gum, lozenge or inhaler) that allow intermittent dosing are preferred for women with low levels of nicotine addiction or who have successfully reduced smoking and women who have quit smoking using NRT patch for several weeks
Remove NRT patch at night
Combination of NRT patch and short acting NRT is recommended
**Bupropion**
Not recommended for use in pregnant or breastfeeding women
There is limited experience from the use of bupropion, may be a reasonable treatment option for pregnant women who are unable to quit smoking
**Varenicline**
Not recommended for use in pregnancy or breastfeeding women

Until recently the use of NRT among pregnant women was considered a second line therapy for those unable to quit with counseling alone, as concerns had been raised about the possible effect of nicotine itself on the fetus^1,38,39^. Relative to smoking, blood nicotine levels are lower when using NRT, nicotine is delivered more slowly, and exposure to the harmful substances contained in tobacco smoke, in particular CO, is avoided^29,40,41^. Although evidence, from the only trial to have followed infants after birth, suggests that the use of NRT actually promotes healthy developmental outcomes in infants, especially if quitting takes place during the first weeks of the second trimester, a period which is very important to the development of the fetus. The last Cochrane review concludes that there is no evidence that NRT used for smoking cessation in pregnancy has either positive or negative impacts on birth outcomes^1,40^. Current opinion is that the use of NRTs in pregnancy carries a small potential risk to the health of the fetus and that using NRT is actually far safer than smoking while being pregnant^11-13^. Further research evidence on NRT safety is needed, ideally from placebo-controlled RCTs that achieve higher adherence rates, monitor infants’ outcomes into childhood, and investigate higher doses of NRT than those tested in the included studies^1^.

The efficacy of NRT in supporting cessation during pregnancy has been mixed but overall its use is favored as a smoking cessation aid in pregnancy^1,13,34,42^. A recent Cochrane review found the use of NRT in pregnancy increased rates of smoking cessation, measured in late pregnancy, by approximately 40%^42^. However, the same review found efficacy is not as evident when evidence from only randomized controlled trials (RCTs) is examined^42^. The fact that most trials have reported a low adherence to NRT by participants limits our ability to fully understand the potential effectiveness of NRT in supporting cessation^1,42-44^. The lack of NRT efficacy, noted in pregnancy compared with ‘non-pregnancy’ NRT efficacy, could possibly be explained by the increased metabolism of nicotine in pregnancy and the low adherence^1,29^. Further evidence in terms of the efficacy of NRTs, ideally from placebo-controlled RCTs with high rates of compliance with NRT treatment, is needed in order to fully understand the role of NRT in supporting cessation during pregnancy^1^. Current guidance from the American College of Obstetricians and Gynecologists, ENSP, NICE, IPCRG and NCSCT, based on available evidence, is that pregnant women who have not been able to quit smoking can use NRTs to support cessation, provided they have been informed of the risks and benefits^11-13,45,46^.

Until recently, guidance has been conservative in terms of dosing of NRT, with clinical guidelines recommending that short-acting NRT products, such as NRT gum, be used rather than the long-acting NRT patch^47^. However, newer evidence suggests that higher dosing of NRT may be required to support cessation in particular among women with higher rates of addiction and/or significant cravings^42^. Moreover, due to the increased metabolism of nicotine during pregnancy, NRT can become less effective at lower doses and such guidance around conservative dosing may be inappropriate^1,29,42^. As is the recommendation in the general population of smokers, there is also practical experience that the combination of the NRT patch and a short-acting NRT (i.e. gum, inhaler, lozenge) benefit pregnant women^9,11,12,44,48^. Current expert opinion recommends the use of the NRT patch during the day in combination with a short-acting oral NRT product and removing the patch at night if a woman does not typically smoke at night^9^. The use of short-acting NRT, which provides intermittent dosing, as a monotherapy is recommended for women with lower levels of nicotine addiction, or women who have been successful in cutting back on smoking but have not been able to quit^12,13,42,44,48,49^. Investigating higher doses of NRT than those tested in the included studies would be ethical for future RCTs^1^.

Due to the lack of safety and efficacy research among pregnant women, neither varenicline nor bupropion is recommended for use during pregnancy or among breastfeeding women^9,12,13,21,29,37,39,50,51^. There are reports on current use of bupropion for cessation among pregnant women from some cessation centers, however the available evidence is still of poor quality^8,11,52,53^. A 2017 review identified eight studies that reported on birth outcomes among women using bupropion during pregnancy^52,53^. Bupropion’s use in the first trimester was associated with a small elevation in cardiovascular defects and results were confounded^52^. There has been only one placebo-controlled RCT investigating bupropion, which experienced recruitment challenges and randomized only 11 women^53^. A second review that reported on 14 studies found no strong evidence of positive or negative outcomes associated with the use of bupropion. Published reviews, to date, have found no evidence that treatment might be harmful relative to the risk of continued smoking, however additional evidence is required to inform practice^11,12,52,53^. Varenicline’s safety has been evaluated from a small number of studies that did not find evidence of teratogenicity but data are limited^54^; it is thus not recommended in pregnancy or among breast feeding _women_^9,11,12,34,45,46,54,55^.

### Digital interventions

Women of reproductive age and particularly pregnant women underutilize evidence-based smoking cessation services such as counseling and quit lines. Digital interventions including mobile health (mHealth) interventions may offer an innovative method for providing evidence-based smoking cessation support to a population that is otherwise difficult to reach. A meta-analysis of digital interventions to support smoking cessation in pregnancy found computer-based (OR=3.06, 95% CI: 1.28–7.33) and text-message (OR=1.59, 95%CI: 1.07–2.38) interventions were the most effective digital platforms when compared to control groups^56^. The review found that text messages should be tailored to the individual smoker and use the following behavioral techniques: giving information about the consequences of smoking and what to expect when trying to quit, encouraging and boosting self-efficacy, and motivating and giving reminders of how to deal with difficult situations^56^. There is some evidence that an engaging, structured digital intervention that is highly tailored and targeted to pregnancy, and additionally combined with personal contact, may be attractive to pregnant smokers seeking help online^27^. Further research is required to fully understand the role of digital interventions in supporting cessation in pregnancy.

### Electronic cigarettes

The promotion of e-cigarette use as a safer alternative to cigarette smoking has led to increased use, even in pregnancy. The United Kingdom, in particular, has indicated support for the use of e-cigarettes as a second-line therapy for pregnant women who smoke; stressing that licensed NRT products are the preferred option; women who choose to use e-cigarette during pregnancy should not be discouraged from doing so if it helps her to stay smoke free^13,34,47,57-59^. Outside UK, societies like the European Respiratory Society do not encourage the use of e-cigarettes^12,45,60^.

There is currently insufficient research on the effects of e-cigarette use on the fetus. While e-cigarettes are not risk-free, they do not expose users to the harmful effects of CO due to the absence of combustion and are as such possibly of lower risk than smoking cigarettes^58,59^. However, studies have found that the nicotine consumed by e-cigarettes is similar to that consumed by cigarette smoking^58^. Experts note that it is unclear at present if nicotine intake via e-cigarette is safe during pregnancy^2^. In fact, a study by Bahl et al.^61^, which evaluated different e-cigarette refill fluids, found all flavors except one to be cytotoxic to human embryonic stem cells. Given the fact that there is currently insufficient research on the effects of e-cigarette use on the fetus and its role as a cessation aid, it is not prudent to recommend e-cigarette use during pregnancy at this time^2^. More research is required to inform clinical practice in terms of e-cigarette use in pregnancy and potential effects on pregnancy outcomes^58,60^.

### Pregnant women who are not ready or motivated to quit smoking

There will always be pregnant smokers who refuse to go through the process of trying to quit, either under the impression that the risks do not apply to them or not even disclosing the true reasons behind their reluctance. For women not motivated to quit smoking, counseling should focus on enhancing motivation and addressing the woman’s personal barriers to cessation.

The 5Rs (Relevance, Risks, Rewards, Roadblocks, Repetition) process can be used (Table 4)^4,12^. The use of carbon monoxide screening or monitoring can be an effective non-judgmental way of identifying maternal exposure to tobacco smoke that might not otherwise be discussed^9,62^. MI (described above) is also a counseling technique, which can be used with pregnant women who are ambivalent or resistant to change.

**Table 4:** The 5Rs

*The 5Rs*	
**Relevance**	Smoker identifies motivational factors
**Risks**	Smoker identifies potential negative consequences of continued smoking
**Rewards**	Smoker describes how quitting would benefit her and her family
**Roadblocks**	Smoker identifies barriers to quitting
**Repetition**	Repeat at every visit for patients who smoke

### Addressing secondhand smoke

Healthcare professionals should provide pregnant women, their partners, and their other close family members with consistent advice and information about the existing risks of exposure to SHS, as well as effective strategies in order to reduce exposure, possible alternatives, and offer smoking cessation _support_^3,9,51,63^.

Only a smoke-free environment will promote optimal perinatal health for the woman and her fetus or newborn. It is therefore imperative for all healthcare facilities, all work places, and all public places to be smoke-free in order to promote the protection of all people, especially pregnant women^10^. Countries that have enforced a smoke-free legislation are evidently enjoying reduced stillbirth and neonatal death rates^10^; this legislation should be considered for adoption across the whole world in order to achieve more positive perinatal outcomes.

### Preventing a relapse in the post-partum period

Between 47% and 63% of women who manage to quit smoking during pregnancy will unfortunately relapse within the first 6 months following birth^3,10,63-65^. Relapse to smoking is also associated with lower rates of breastfeeding among women^66^. In fact, some new mothers prefer not to breastfeed their newborns in order to resume smoking^5,7,66,67^.

There are many reasons why women return to smoking during the post-partum period (Table 5). For many women the motivation to quit smoking was specific to the health of their unborn baby, so that following birth with this risk no longer present, many women no longer feel the same level of motivation to stay smoke-free. Additionally, strong social pressures to remain smoke-free when pregnant are no longer factors in the postpartum period and women may not be exposed to common smoking triggers such as alcohol and caffeine during the antenatal period. Weight concerns, return of triggers (e.g. alcohol, caffeine), smoking spouse, underdeveloped coping strategies or overconfidence, less social pressure to stay abstinent, sleep deprivation, financial worries, increased stress (relationship troubles, medical problems, stressful events) are some of the reasons why women return to smoking^5,64,67^.

**Table 5:** Causes of relapse

*Causes of relapse*
Weight concerns
Nostalgia for former self, for a happier, less stressful time
‘Controlling’ smoking
Return of triggers (alcohol, caffeine)
Underdeveloped coping strategies and overconfidence
Never really having quit
Smoking spouse
Less social pressure to stay quit
Increased stress (relationship troubles, medical problems, stressful events etc.)
Sleep deprivation
Financial worries

Adapted from Quinn et al.^64^ and from Ashford et al.^66^.

It has been shown that women who quit, having received support from a healthcare professional trained in smoking cessation, have a better chance of not relapsing^7,63,67^. As such, maternity care providers should receive training in tobacco treatment delivery and be prepared to intervene. Specifically, this will include discussing the dangers of first-hand or secondhand tobacco smoke, both at antenatal visits as well as visits in the postpartum period, and encouraging not only smoke-free environments in the home but also supporting the maintenance of smoking abstinence. Health care professionals should discuss in the third trimester of pregnancy the risks, to increase the patient’s awareness of the potential for relapse, reaffirm her commitment to abstinence and begin to change the motivation for quitting from extrinsic sources to intrinsic ones.

Research to date, examining strategies employed either during pregnancy or postnatally to assist women to avoid a possible relapse, has not been very effective and there is a need for further research to inform practice in this area^57^. Providing education on the dangers and the potential harm that SHS can have on the health of infants, has been shown by itself to be a considerable motivating factor for many women not to return to smoking following birth as they generally take the health of their infants into serious consideration^57^. A prolonged period of breastfeeding is also a factor contributing to women remaining smoke-free and avoiding a potential relapse postnatally^3,7,67^. Examples of other helpful messages include information on behavioral and mental coping skills, exercises regarding triggers to smoke, reminders of why they quit, emphasizing the negative health effects for both mother and baby, including effects of SHS exposure, information on weight gain, and ways they can spend the money they save by not buying cigarettes.

Hospitalization of an infant or a mother after birth can also be a good opportunity for identifying mothers or family members who have recently quit smoking and to intensify the consulting support provided to them, in order to prevent them from relapsing. It has been found that during their hospitalization, women are generally more sensitive to receiving smoking cessation counseling services^12^.

### Addressing tobacco use in maternity care settings

Given the importance of tobacco use on pregnancy outcomes, all the healthcare professionals, especially obstetricians and midwives, must be prepared to identify and support pregnant smokers with quitting using evidence-based treatments as a standard of care in maternity care settings^3,5,11,12,46,48^.

### The 5As or 3As approach

**Figure 1:**
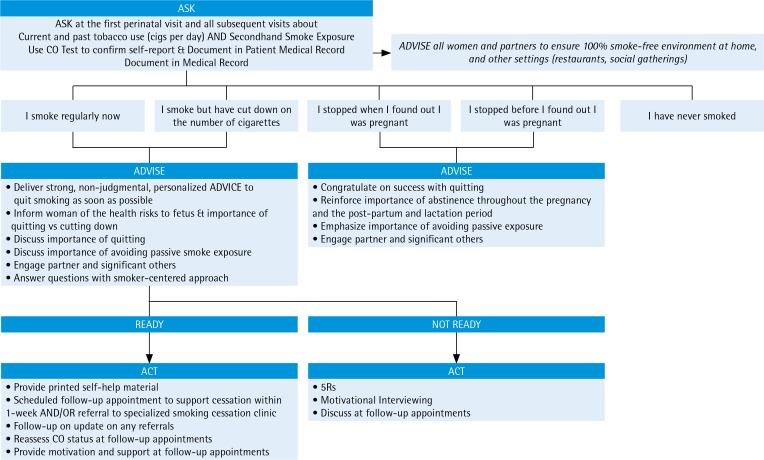
3As approach for smoking cessation in maternal health settings

The recommended sequence for delivering tobacco treatment interventions in clinical settings, including maternal care settings, is known as the 5As approach. The 5As (Ask, Advise, Assess, Assist, and Arrange for follow-up) should be used at the first antenatal appointment and during subsequent appointments^4,11,30^. The approach can also be shortened to ‘Very Brief Advice’ or the 3As (Ask, Advice, Act)^4,11,12^. The emphasis for the 3As model is for the maternal health team to ask and document the smoking status of all pregnant women, to provide brief advice to women about the importance of smoking cessation and act to support cessation by referring pregnant women who smoke to available quit-smoking services either within the maternity care team or community who will then provide evidence-based quit smoking assessment, assistance, and follow-up (Figure 1). We review here the key elements of the 5As delivery in maternity care settings.

#### Ask

All pregnant women should be assessed for both personal tobacco use as well as exposure to SHS every antenatal appointment^4,11,12^. The validity of self-reported smoking information must be of special concern as some women may not feel comfortable revealing that they are smoking during pregnancy and could therefore be missing out on the opportunity to receive help with quitting^3,12,25,57^. It is estimated that up to a quarter of women who smoke, deny it when asked by their maternity care provider^3,12,25,57^. The use of multiple-choice questions in survey questionnaires, rather than simple yes/no questions, can increase the possibility of getting accurate feedback about the real smoking status of women by as much as 40%^57^. Specifically, pregnant women can be asked to choose what answer describes best their smoking status^4,11,12,46^, as in the following:

- I have never smoked or have smoked <100 cigarettes in my lifetime;
- I stopped smoking before I found out I was pregnant, and I am not smoking now;
- I stopped smoking after I found out I was pregnant, and I am not smoking now;
- I smoke some now, but I have cut down on the number of cigarettes I smoke since I found out I was pregnant; and
- I smoke regularly now, about the same as before I found out I was pregnant.

The use of CO monitors when available is a recommended practice for obtaining an accurate report of maternal tobacco use^4,11-13,25,46,51^. In addition to being a good practice, the CO test will allow women to see a practical measurement both of their own smoking status and can serve as an intervention for motivating smoking cessation. During pregnancy CO levels of <4 ppm are considered to be that of a non-smoker^57,68^. In the event that CO ≥4 ppm and the woman denies being a smoker then this should be further explored. It is important that the results of CO testing be introduced in a sensitive way in order to minimize any embarrassment^9,57,68^. Additionally, women should also be asked if their partner and family are also smokers and if they smoke in the home or car, in order to assess SHS exposure.

#### Advise

A non-judgmental and supportive approach has been shown to be particularly important in supporting cessation during pregnancy and should be adopted by all maternity care professionals^28,30,69^. Maternity care providers should stress the benefits of quitting and emphasize the impact of smoking on both the woman and fetus using clear, strong and personalized non-judgmental language. Since a considerable number of pregnant women are actually unaware or underestimate the risk associated with smoking in pregnancy, both to the fetus and to themselves, this information should be provided in a clear and personalized manner^28,30,69^. The direct effects of CO on the placenta and fetus should be explained^3,4,11,30,45,46^, and if available, the results of the woman’s CO test can be used to provide tangible feedback of the effects of smoking on the fetus and enhance the pregnant woman’s knowledge of the adverse effects of tobacco use on the fetus growth and development^46^. Verbal advice should be enhanced through leaflets and other reading materials.

It should be made clear to all women that there is no safe level of smoking during pregnancy. Quitting smoking entirely should always be advised instead of just reducing daily cigarette consumption, since a reduced number of cigarettes is not equivalent to a reduced health risk^4,11,12,45,46^.

If the partner or other close family members smoke, they should also receive advice in terms of the risk of SHS during pregnancy and offered support with quitting^4,5,11,12,46^. Given the strong relationship with a pregnant woman’s ability to quit smoking, the woman’s partner and other significant family members who smoke should also be encouraged to support and not to undermine a woman’s cessation efforts both during pregnancy and in the postpartum period. Family members or those who spend a lot of time with the pregnant woman should be encouraged to assist with ensuring the home and car are 100% smoke-free. The arrival of the new baby can be also an opportunity for partners and other family members to quit smoking^4,5,11,12,46^.

#### Assess

The woman’s readiness to quit, her dependence on nicotine, as well as her confidence and concerns about quitting should be assessed. If the pregnant smoker declares willingness to try to quit, then healthcare professionals should move to the actions described in the following two steps (Assist and Arrange for follow-up). Three other parameters should be assessed^58^:

1. On a scale from 0–10, what is your intention to quit smoking for good (0=Quit only for the pregnancy, 5=Not sure, 10=Quit forever)
2. On a scale from 0–10, how important is it to you to quit smoking? (0=Not important at all, 5=Not sure, 10=Very important)
3. On a scale from 0–10, if you were to try to quit smoking, how confident do you feel that you would be able to do it? (0=Not confident at all, 5=Not sure, 10=Very confident)

If the pregnant woman declares she is not ready to quit smoking, then counseling should focus on exploring barriers to quitting and increasing her motivation to quit smoking^4,5,11,12,46^. However, if the pregnant woman clearly declares being unwilling to make any sort of quitting attempt at that time, her interest should be reassessed at subsequent appointments.

#### Assist

Support with quit smoking should be provided to all women who smoke or have recently quit smoking^57^. Assistance with quitting should include behavioral counseling, an action plan, and for some women the use of NRT as summarized above. Members of the maternity care team including obstetricians, midwives and other healthcare professionals should be prepared to provide at minimum brief counseling to all women who smoke or have recently stopped smoking and who are planning a pregnancy^4,5,11,12,46^. They should also be helped to cope with other smokers in their social environment by encouraging them to quit as well, or by kindly requesting that they do not smoke in their presence^12^. Providing smoking cessation support has not been found to cause additional measurable psychological stress to women, contrary to what was believed by healthcare professionals in the past^4,5,11,12,46^.

#### Arrange follow-up

Where available, pregnant women who smoke should be referred to a specialized smoking cessation service delivered by community or maternity care specialists in order for them to receive more specialized counseling on their quit attempt. The local smoking cessation helpline number should be provided, where available. Placing a call to the helpline during an appointment could be an effective way for it to be introduced. Follow-up appointments should always be arranged in order to ensure that progress is being made^4,5,11,12,46^. At the first follow-up appointment, pregnant smokers should be asked if they have contacted any of the suggested smoking cessation services. If these services have already been contacted and initial support has been received, then the arrangement of succeeding appointments with the services should be offered, in an attempt to encourage the progress of the cessation attempt^4,5,11,12,46^. If none of the suggested smoking cessation services has been contacted, then pregnant smokers should be asked if they feel prepared to maybe contact one now, thus encouraging them to eventually start their quit attempt. Additionally, in the event that no service has been contacted and all offers for help have firmly been declined, then the smoker’s decision must be accepted without adopting a critical attitude and the fact that the offer to help quit smoking is always open must be explained.

## CONCLUSIONS

Maternal tobacco use and exposure to secondhand tobacco smoking during the prenatal and postnatal periods cause a plethora of negative effects on the fetus vital organs and systems and on the mother. Most women recognize a general risk, but not the magnitude or specifics. Due to the stigma related to tobacco use, many women under-report smoking during pregnancy, which can be a barrier to treatment. There may be significant barriers to quitting during pregnancy and particularly in the post-partum period. There have been several recent updates to clinical practice regarding the treatment of tobacco use in pregnancy, it is important for the latest guidance to be put into practice in all maternity care settings, in order to decrease the percentage of pregnant smokers. Effective treatment requires addressing barriers to quitting, the provision of counseling at regular intervals throughout the course of the pregnancy and into the post-partum period, as well as the provision of NRT for smokers who may have higher rates of nicotine addiction or are unable to quit with counseling alone. Obstetricians, midwives, family physicians, and other healthcare professionals, should be trained in smoking cessation and in behavioral counseling, and be prepared to intervene with pregnant women who smoke.

## CONFLICTS OF INTEREST

The authors declare that they have no competing interests, financial or otherwise, related to the current work. S. Papadakis reports grants from Global Bridges (Pfizer Education and Change), outside the submitted work. The rest of the authors have also completed and submitted an ICMJE form for disclosure of potential conflicts of interest.
